# Presumed filter-feeding in a deep-sea benthic shrimp (Decapoda, Caridea, Stylodactylidae), with records of the deepest occurrence of carideans

**DOI:** 10.3897/zookeys.646.10969

**Published:** 2017-01-17

**Authors:** Mary Wicksten, Sammy De Grave, Scott France, Christopher Kelley

**Affiliations:** 1 Department of Biology, Texas A&M University, College Station Texas 77843-3258, USA; 2 Oxford University Museum of Natural History, Parks Road, Oxford, OX1 3PW, United Kingdom; 3 Department of Biology, University of Louisiana at Lafayette, P.O. Box 43602, Lafayette, Louisiana, 70504 USA; 4 Hawaii Undersea Research Laboratory, University of Hawaii, Honolulu, Hawaii 96822 USA

**Keywords:** Deep-sea shrimp, Stylodactylidae, feeding, carideans, Marianas Trench

## Abstract

Using the remotely operated vehicle *Deep Discoverer*, we observed a large stylodactylid shrimp resting on a sedimented sea floor at 4826 m in the Marianas Trench Marine National Monument. The shrimp was not collected but most closely resembled *Bathystylodactylus bathyalis*, known previously only from a single broken specimen. Video footage shows the shrimp facing into the current and extending its upraised and fringed first and second pereopods, presumably capturing passing particles. The video footage is the first ever to show a living deep-sea stylodactylid and constitutes the deepest record for the family. We provide a list of the deepest reports of caridean shrimps world-wide.

## Introduction

Benthic caridean shrimps living at 1000 m or more are poorly known, for the most part represented by specimens taken by trawls. To the best of our knowledge, none of them has been brought to the surface alive. Often, the fragile pereopods and antennae are broken or torn off. The feeding modes and form of the appendages of vent shrimps (family Alvinocarididae) have been studied in detail (Komai and Segonzac 2008). Other carideans living on mud or hard substrates away from vents are poorly studied. It is difficult to maneuver collecting equipment among rocks without entanglement. Shrimps living on mud may be difficult to see because they burrow into the sediment or dart away if disturbed. In situ photographs often do not show the shrimp in sufficient detail for identification.

There are three species of *Bathystylodactylus*, the deepest known members of the family Stylodactylidae. These are known from six specimens: one each of *Bathystylodactylus inflatus* from off Taiwan and *Bathystylodactylus bathyalis* from the Coral Sea and four of *Bathystylodactylus echinus* from the eastern Pacific (Hanamura and Takeda 1996; Cleva 1994; Wicksten and Martin 2004). All known specimens are damaged, missing at least some of the appendages. We report here on a living shrimp photographed in situ, provide the deepest report of a stylodactylid shrimp to date, and compare the depth records of the deepest known carideans.

## Methods

We obtained photographs and video of the shrimp from the U.S. National Oceanographic and Atmospheric Administration (NOAA)
Ship *Okeanos Explorer*
(OkEx), administered through the Office of Exploration and Research. The ship is equipped with a 6,000 m-rated dual-body system comprising the remotely-operated vehicle (ROV) *Deep Discoverer* (*D2*) and *Seirios* camera sled. During operations, *Seirios* is tethered to the ship with a standard oceanographic armored, fiber-optic cable (1.73 cm diameter) and *D2* is linked to *Seirios* with a neutrally buoyant tether, thus isolating the ROV from the *OkEx* surface motion to allow precise maneuvering and steady imaging of deep-sea communities.

The ROV *D2* is outfitted with two maneuverable and four fixed video cameras; scientific observations are made primarily using two high-definition video cameras. Light is supplied by 26 LED lamps (195000 lumens total), with eight of these on four hydraulically positioned booms. Paired lasers (10 cm apart) mounted on the fixed, high-definition video camera provide size scale in the imagery. The ROV traversed the seafloor at a speed of approximately ~ 0.1– 0.3 knots (1 knot = 0.514 m s^-1^) with the cameras generally set on wide-angle view, and zooms were initiated to obtain detailed imagery when objects of scientific or aesthetic interest were encountered. High-definition video was transmitted from the *D2* in HD-SDI 1080i format. *OkEx* is equipped with high-speed communication capabilities to enable scientists on the shore to participate in ship operations in time via telepresence (see http://oceanexplorer.noaa.gov/okeanos/collaboration-tools/welcome.html). Scientists (and anyone with an Internet connection) are able to observe live video feeds from the ROV *D2* and participate in real-time via a private Internet chat room and satellite teleconference line.

## Results

From 20 April to 10 July, 2016, the *OkEx* was engaged in the “2016 Exploration of the Marianas” Expedition, a baseline study of deep-water environments in and around the Commonwealth of the Northern Mariana Islands and the Mariana Trench Marine National Monument (MTMNM). *D2* Dive 13 of Leg 3 (30 June 2016) of this expedition explored a site informally named “Twin Peaks” on a seamount in the MTMNM. The *D2* spent 4 hours 48 min traversing the bottom, from 4840 meters depth upslope to 4787 m. The seafloor was mostly thickly sedimented with clay-like particles that were easily disturbed into clouds by the ROV thrusters; small outcroppings of sedimentary rocks were seen frequently as well as occasional large boulders.

Approximately 2 hours 17 min after the ROV reached the bottom and the benthic exploration began, an observation was made of a single individual stylodactylid shrimp (estimated total length 120 mm) at 4826 m depth (21.41774° N, 145.89294° E). No other stylodactylids were observed during the dive or on other dives during this expedition. Other benthic shrimps (Superfamily Penaeoidea) and thread-leg shrimps (Caridea: Nematocarcinidae) were observed on soft substrates, as well as other carideans associated with soft corals (Order Gorgonacea) and sponges (Hexactinellida). When initially observed, the shrimp was facing away from the ROV camera. Participating scientists noted this was an atypical shrimp species for the expedition, so the ROV settled to capture detailed images. After a couple of minutes the ROV was repositioned to get a lateral view of the shrimp. During the approximately 4 minutes of observation (including the ROV maneuver) the shrimp did not move from its initial position on the bottom, facing into the weak boundary layer current. The following environmental data were recorded during the observation: temperature 1.47236°C, salinity 34.69294 PSU, dissolved oxygen 4.96223 mg/L.

With anterior legs upraised, the shrimp faced into the current, presumably using its legs as a net to capture passing particles. The shrimp had first and second pereopods fringed with setae and with extremely slender chelae, characteristic of the family Stylodactylidae (Cleva 1990). The deepest species, belonging to the genus *Bathystylodactylus*, have a long toothed rostrum, eyes without pigment, and a carapace that is pubescent or studded with minute spinules (Hanamura and Takeda 1996, Wicksten and Martin 2004). The shrimp in the video appeared to most closely resemble *Bathystylodactylus bathyalis*, previously only known from a single broken specimen collected at 3515–3502 m in the Coral Sea (Cleva 1994). The present video footage is the first for any of the deep-water dwelling Stylodactylidae. Other invertebrates in the area included a large long-legged isopod (family Munnopsidae), an enteropneust (phylum Hemichordata), a swimming holothurian (Echinodermata), and a hermit crab (*Parapagurus* sp.) with an associated commensal sea anemone (Actiniaria). The ROV was not equipped with collecting equipment that could capture any of these animals and so their species identification remains uncertain.

As in the euphausiaceans, the long, setose legs of the stylodactylid seem to form a “filter basket” that captures particles. The shrimp in the video was not seen to open and expel water by means of pumping, as can euphausiaceans, but instead relied on passive transfer of food particles by the boundary layer current. No other group of carideans is known to feed in this manner (Wicksten 2010). Other decapods filter-feed by means of long feathery antennae, as in the hermit crabs of the genus *Paguritta* and the mole crabs, family Hippidae; or setose third maxillipeds, as in crabs of the family Porcellanidae (Riisgard and Larsen 2010).

## Discussion

**Figure 1.**
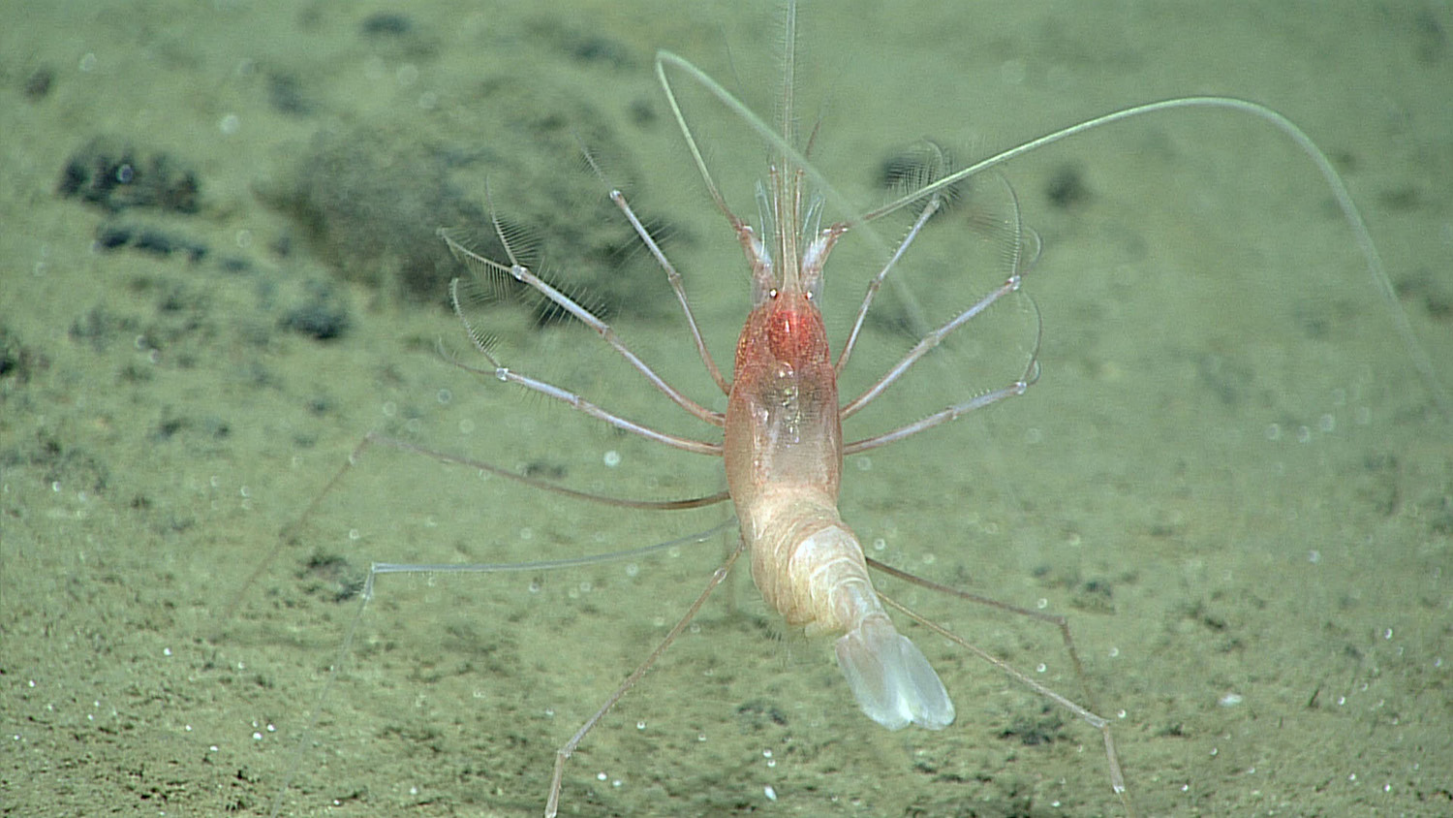
*Bathystylodactylus* cf. *bathyalis*, 4826 m, in situ. Photo extracted from high-definition video captured using ROV *Deep Discoverer*. (Image courtesy of the NOAA Office of Ocean Exploration and Research 2016 Deepwater Exploration of the Marianas).

Relatively few caridean shrimps live at bathyal depths, i.e. greater than 1000 m, with far fewer recorded at abyssal depths of 3000 m and deeper. As well as species of the Stylodactylidae, caridean shrimps reported from abyssal depths include species of the families Bythocarididae, Crangonidae, Disciadidae, Nematocarcinidae, Oplophoridae, Pandalidae and Pasiphaeidae (Table 1). The present observation of *Bathystylodactylus* sp. is the deepest report of a member of this family. The oplophorid *Heterogenys microphthalma* has been collected in trawls as deep as 5060 m (Crosnier 1987). Jamieson et al. (2009) included photographs of “*Acanthephyra* sp.” from baited traps as deep as 6890 m in the Kermadec Trench, but their photographs (Fig. 1B, C) show a shrimp with a short upturned rostrum, characteristic of *Heterogenys microphthalma*. If confirmed this would be the deepest recorded caridean shrimp record, a record currently held by *Glyphocrangon atlantica* at 6364–6373 m (Holthuis 1971).

**Table 1.** Deepest family level records for caridean shrimp species living at depths bathyal and abyssal depths. Systematics follows De Grave and Fransen (2011), with modifications in De Grave et al. (2014, 2015). Depths as well as sampling or observation method are given.

Acanthephyridae
*Acanthephyra quadrispinosa* Kemp, 1939: Crosnier (1987), 5040–5060 m, trawl.
*Heterogenys microphthalma* (Smith, 1885): Crosnier (1987), 5040–5060 m, trawl.
Alvinocarididae
*Rimicaris hybisae* Nye, Copley & Plouviez, 2012: 4960 m, TV grab and slurp gun.
Bathypalaemonellidae
*Bathypalaemonella serratipalma* Pequegnat, 1970: Cleva (2001), 2660–2750 m, beam trawl.
Bresiliidae
*Bresilia pacifica* Hendrickx, 2014: 2010–2046 m, benthic sledge.
Bythocarididae
*Bythocaris cryonesus* Bowman & Manning, 1973: 3803 m, minnow trap.
Crangonidae
*Placopsicrangon formosa* Komai & Chan, 2009: 4807–4824, beam trawl.
Disciadidae
*Lucaya bigelowi* Chace, 1939: 4773 m, open net.
Glyphocrangonidae
*Glyphocrangon atlantica* Chace, 1939: Holthuis (1971), 6364–6373 m, collecting method not specified.
Hippolytidae
*Leontocaris amplectipes* Bruce, 1990: Ahyong (2010), 2182–2119 m, sled.
Nematocarcinidae
*Nematocarcinus challengeri* Burukovsky, 2006: 5477 m, trawl.
Oplophoridae
*Systellaspis debilis* (A. Milne Edwards, 1881): Crosnier (1987), 4987–5025 m, trawl.
Palaemonidae
*Periclimenes pholeter* Holthuis, 1973: Bruce (2011), 2148 m, collecting method not specified.
Pandalidae
*Stylopandalus richardi* (Coutière, 1905): Hayashi and Miyake (1969), 3600 m, midwater trawl.
Pasiphaeidae
*Parapasiphae compta* Smith, 1884: Crosnier (1988), 4990 m, trawl.
Physetocarididae
*Physetocaris microphthalma* Chace, 1940: Wasmer (1985), 1200–2200 m, non-closing trawl.
Psalidopodidae
*Psalidopus tosaensis* Toriyama & Horikawa, 1993: 2765–2881 m, beam trawl.
Stylodactylidae
*Bathystylodactylus* sp.: present report, 4820 m, video observation.
Thoridae
*Lebbeus laurentae* Wicksten, 2010: Komai et al. (2012), 2640 m, slurp gun.

Video link: http://oceanexplorer.noaa.gov/okeanos/explorations/ex1605/dailyupdates/media/video/0630-mudmonsters/0630-mudmonsters.html
